# Is Laparoscopic Pyeloplasty for Ureteropelvic Junction Obstruction in Infants Under 1 Year of Age a Good Option?

**DOI:** 10.3389/fped.2019.00352

**Published:** 2019-09-25

**Authors:** Corina Zamfir Snykers, Elea De Plaen, Sophie Vermersch, Manuel Lopez, Karim Khelif, Stephane Luyckx, Paul Philippe, Francois Varlet, Henri Steyaert

**Affiliations:** ^1^Hôpital Universitaire Des Enfants Reine Fabiola, Université Libre de Bruxelles (ULB), Brussels, Belgium; ^2^Centre Hospitalier Universitaire de Saint-Étienne, Saint-Étienne, France; ^3^Centre Hospitalier de Luxembourg, Luxembourg, Luxembourg

**Keywords:** laparoscopic pyeloplasty, ureteropelvic junction obstruction, hydronephrosis, renal function, infants, children under 1 year of age

## Abstract

**Purpose:** Laparoscopic pyeloplasty in children younger than 1 year of age is still debatable due to its supposed technical difficulties and failure rate. We present our experience and outcome in infants.

**Materials and Methods:** A retrospective study was conducted in 3 Departments of Pediatric Surgery. We reviewed the records of the children under 1 year of age operated on for ureteropelvic junction obstruction (UPJO), between 2007 and 2017. Anderson-Hynes laparoscopic transabdominal dismembered pyeloplasty was performed. Patients' demographics, results of preoperative and postoperative exams, perioperative details, complications, hospital stay, and long-term follow-up results were analyzed.

**Results:** Sixty cases were operated on during this period (49 boys, 11 girls). Mean age at operation was 4.5 months (1–12 months). Mean operating time was 140 min (80–240 min). There was no conversion in this group. There were four early complications: 1 ileus, 1 hypertension immediately post-operatively requiring medical treatment, 1 omental herniation through a drain orifice, and 1 percutaneous transanastomotic stent migrated intra-abdominally. The two last children had to be reoperated. Mean hospital stay was of 2 days (1–10 days). Late complications: two patients (3.4%) presented a recurrence of UPJO, one had been re-operated 15 months later and for the patient with persistent hypertension, nephropexy was performed for malrotated kidney, 1 year after pyeloplasty. Long term follow-up with a mean of 2.8 years (1–10 years) showed that surgery improved mean pelvic dilatation from 31.8 mm (13–63 mm) preoperatively to 15.3 mm (4–40 mm) postoperatively (*P* < 0.0001). The renal function slightly improved, from a mean of 35.7% (5–55%) it passed to 40.5% (0–54%), *p* = 0.137. In three cases the operated kidney became finally non-functional and atrophic.

**Conclusions:** Laparoscopic transperitoneal pyeloplasty is feasible and safe in children younger than 1 year of age. Nevertheless, it requires experience and good intra-abdominal suturing skills. Laparoscopic pyeloplasty has a success rate comparable with open treatment but with less morbidity and better cosmetic results.

## Introduction

Prenatal diagnosis improved the detection of kidney anomalies during pregnancy. This allows early confirmation of diagnosis and regularly follow-up postnatally. In ureteropelvic junction obstruction (UPJO), when necessary, surgery is performed under 1 year of age, in order to save renal function.

In UPJO, the standard surgical technique is Anderson-Hynes pyeloplasty, done by open or by laparoscopic surgery. Laparoscopic pyeloplasty was described in 1995 by Peters (1). The laparoscopic approach has gained more and more popularity in the last years in older children. However, in the young and very young (1–2 years old) still remains a matter of debate. There are only few studies that show the results and advantages of laparoscopy in this age-group (2–4). In infants, the working space is smaller and requires good skills and experience in laparoscopic surgery. In order to assess the feasibility and outcomes of laparoscopic pyeloplasty in infants we reviewed our results in three centers with experimented surgeons.

## Materials and Methods

A retrospective study of patients below 1 year of age operated on for UPJO between 2007 and 2017 was conducted in Three Departments of Pediatric Surgery. All patients underwent laparoscopic transperitoneal pyeloplasty according to Anderson-Hynes technic. We focused on patients' demographics, results of preoperative and post-operative studies, perioperative details, complications, hospital stay, and long-term follow-up results.

Ethical review and approval was not required for the study on human participants in accordance with the local legislation and institutional requirements. Written informed consent for participation was not required for this study in accordance with the national legislation and the institutional requirements by the “Hôpital Universitaire Des Enfants Reine Fabiola” ethics committee.

The patients had prenatal diagnosis of pelvic dilatation which had been evaluated according to the classification of the Society for Fetal Urology (5, 6). Postnatal confirmation of UPJO was established by regularly ultrasound and MAG 3 renal isotopic scan. Voiding cystourethrogram was performed, according to each department algorithm.

Indications for surgery were: loss of renal function due to obstruction seen on diuretic renography, worsening of the hydronephrosis on serial ultrasound or/and severe urinary tract infection with evidence of obstruction. In the three departments antibiotics had been administered during surgery. All surgeons had a long experience in laparoscopy. Pyeloplasty was performed using 3 ports, one of 5 mm for the camera placed infra-umbilically and 2 of 3 mm for the instruments, one in the epigastric area and one in the right or left iliac fossa, according to the side of the affected kidney. The anastomosis was made by absorbable 5/0 running sutures in all cases. Stenting was systematically done using either a double J or a percutaneous trans anastomotic stent. In the first patients of this series a drain was left in place. The bladder was drained by a Foley catheter which was removed next day. The post-operative pain control was done by regional caudal block. The percutaneous trans anastomotic stent was withdrawn at 14 days after surgery in the out-patient clinic. Double J stent were cystoscopically removed about 6 weeks after surgery under general anesthesia as day case.

The complications were assed according to Clavien-Dindo classification (7).

Long term follow-up was performed by ultrasound at 3 and 6 months post-operatively and annually after. Renal function was assessed using a MAG3 renography, 6 months after surgery.

All patients who had not at least 1 year of follow-up or were lost after surgery were excluded from the study.

A statistical analysis was performed using Student's *t*-test.

## Results

Sixty patients (49 boys and 11 girls) with a mean age of 4.5 months (1–12 months) were operated on during this period in the three departments (Table 1). Prenatal diagnosis was established in 59 patients. In 38 the hydronephrosis was severe with parenchymal loss (grade IV according to the Society for Fetal Urology). In two patients kidney drainage was decided during pregnancy. In 20 patients the right kidney was affected and in 40 patients the left. Patients' weight varied from 5.2 to 9.5 kg with an average of 7.5 kg. All patients were followed by regularly ultrasound and MAG 3 renal isotopic scan. Voiding cystourethrogram was performed in 43 patients. Associated anomalies were: horseshoe kidney in 1 patient, stenotic megaureter on the same side in 1 patient and contralateral multicystic dysplastic kidney in three patients. Ten patients in the series presented at least one episode of urinary infection before surgery. Associated vesicoureteral reflux was diagnosed in three patients unilateral and in five bilateral.

**Table 1:** Patients' demographics.

Number of patients	60 (49 males/11 females)
Pre-natal diagnostic	59 patients
Age at surgery	4.5 months (1–12 months)
Type of hydronephrosis (SFU)	22 grade III/38 grade IV
Weight at surgery	7.5 kg (5.2–9.5 kg)
Affected side	20 right kidney/41 left kidney
Associated anomalies	1 bilateral UPJO: left side operated at 8 month-old, right side at 3 year-old 1 horseshoe kidney 1 stenotic megaureter on the same side as UPJO 3 contralateral multicystic dysplastic kidneys
Complications before surgery	1 patient: atrial and ventricular septal defect and persistence of arterial duct, nephrostomy at 7 days of life because of severe obstruction; pyeloplasty performed 5 months later after the cardiac surgery 1 patient: pyonephrosis and sepsis had nephrostomy at 10 days of life; surgery was performed 4.5 months later 1 patient: upper pole perforation at 3 months of age required nephrostomy; surgery performed 15 days later
Mean operating time	140 min (80–240 min)
Conversion	0
Stent (Double J/percutaneous transanastomotic)	57 (29 Double J/28 percutaneous transanastomotic)
Suction drain	7

One patient had a bilateral UPJO: left side was operated at 8 months and the right at 3 years of age. In one patient with atrial and ventricular septal defect and persistence of arterial duct a nephrostomy was placed at 7 days of life because of severe obstruction and pyeloplasty was performed 5 months later after the cardiac surgery. One patient required nephrostomy at 10 days of life due to sepsis with pyonephrosis. Surgery was done 4.5 months later. Another patient presented an upper pole perforation at 3 months of age and required also nephrostomy. Surgery was performed 15 days later.

Mean operating time was 140 min (80–240 min). There was no conversion in this group. In three patients polar renal vessels were found. All cases had a pyelo-ureteral stent except three were stenting wasn't possible due to technical difficulties. Twelve of the first cases had also a drain in the operated area (beginning of the experience). In one patient, operated on at 6.5 months, caliceal lithiasis was extracted under direct vision, during surgery. In another patient operated on at 1 month of age due to severe obstruction, pus was found in the pelvis during intervention, requiring prolonged post-operative antibiotherapy.

There were 4 (5%) early post-operative complications (Table 2): 1 ileus (grade II complication according to Clavien-Dindo classification), 1 omental herniation through a trocar orifice (reoperated, grade IIIb), 1 percutanous transanastomotic stent migration intra-abdominally (reoperated, grade III b), 1 episode of hypertension in the first hours after surgery (grade II). This last patient necessitated a long-term antihypertensive treatment.

**Table 2:** Early complications.

**Complications**	**Patients**	**Clavien-Dindo classification**
Prolonged ileus	1	Grade II
Omental herniation	1	Grade IIIb
Percutaneous trasanastomotic stent migration	1	Grade IIIb
HTA	1	Grade II

Patients were discharged between the 1st and 10th post-operative day (mean: 2 days) with antibiotic prophylaxis until stent withdrawal (26 patients) or without (34 patients) according to each department protocol.

Six patients (10%) presented un episode of urinary infection during stenting period. Eleven patients (18.4%) had another urinary infection during long term follow-up. All received an adapted antibiotic therapy.

Two patients (3.4%) presented a recurrence of the UPJO (grade IIIb complications) and were re-operated, using again a laparoscopic approach, 12 and 15 months after the first surgery. One of these patients, is the one who presented a post-operative persistent hypertension requiring long-term antihypertensive treatment. At early follow up, ultrasounds found decreased diameter of the pelvis and on MAG 3 scan, done 6 months after surgery, an improvement of renal function. One year later the control ultrasound showed a new increase of the diameter of the pelvis and obstruction at the emptying of the kidney on renal scan. Exploratory laparoscopy found malrotated kidney, with the pelvis oriented posteriorly, lying on the pyelo-ureteral junction. The malrotation wasn't obvious during the first surgery probably because of the very dilated pelvis which was changing the natural position. Diuretic test showed complete emptying of the pelvis when kidney was lifted. Nephropexy was decided due to the thickness of parenchyma and kidney position. The pelvis was attached to the lateral abdominal wall by three interrupted stitches of non-absorbable suture. The postoperative course was uneventful with quick blood pressure normalization. Control ultrasounds showed a favorable evolution and normal empty on isotopic scan, 6 months after re-do surgery.

**Figure 1:**
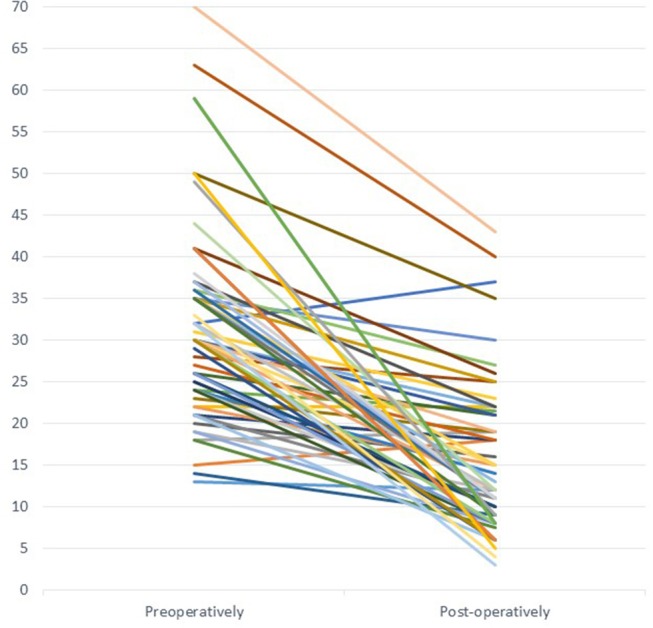
Changes in pelvis diameter after pyeloplasty on ultrasound (*P* < 0.0001).

Long term follow-up with a median of 2.8 years (from 1 to 10 years) showed that surgery had improved mean pelvic dilatation from 31.8 mm (13–63 mm) preoperatively to 15.3 mm (4–40 mm) postoperatively. This is statistically significant *P* < 0.0001 (Figure 1). From functional point of view, there was a slight improvement, from a mean of 35.7% (5–55%) it passed to 40.5% (0–54%), not statistically significant *p* = 0.137. Three out of 60 patients finally lost their operated kidney (5%). We noticed that at longer follow-up, if MAG 3 scan was repeated years later for different reasons, renal function slightly improves by time comparing with the control performed at 6 months post-operatively.

## Discussion

UPJO is the most frequent obstructive uropathy in infants. According to international guidelines and recommendations the indications for surgery are an impaired function of the affected kidney (in particular if impairment is increasing), augmentation of anteroposterior diameter of the pelvis on repeated ultrasound (grade IV dilatation as defined by the Society for Fetal Urology) or severe infection.

Laparoscopic pyeloplasty is increasingly used during the last years, due to the advancement in technological development, standardization of the technique and improved experience of surgeons. Even when laparoscopy has similar results with open surgery, it offers additional advantages like shorter hospital stay, less analgesic medication and better cosmesis. It is a demanding procedure, requiring specific experience, and skills in intracorporeal suturing.

However, it has not been widely approved yet (8–10). Gatti et al. (11) in a prospective randomized controlled study comparing open to laparoscopic pyeloplasties found that the two techniques were comparable and effective methods. The only significant outcomes were a longer operative time in laparoscopic group (139.5 vs. 122.5 min) but a shorter hospital stay (25.9 h in laparoscopic group and 28.2 h in open group). But it excludes the infants under 1 year of age. The conclusion is that the surgical approach should be based on family preference for incision aesthetics and surgeon comfort. In their discussion the authors admitted that the study didn't address to the amount of analgesic used at home and the time needed to return to school activities, which could be longer in patients with flank incision. This is another major issue to be discussed when laparoscopy is compared to open surgery. The scar grows with age and could change the appearance, sometimes becoming keloid. In his Editorial comment to this study, Whittam (12) stressed these points and rose the question if the new techniques like laparoscopy did not push on older techniques to improve their own recovery time and cosmesis?

In infants, some surgeons prefer to perform pyeloplasty by a posterior approach through a small horizontal incision with patient in prone position. They combine the advantage of a small incision with good view of the UPJO.

While certain authors advocate the advantages of retroperitoneoscopic pyeloplasty (13–15) others plea for transperitoneal laparoscopic approach because of larger working space for suturing, shorter operative time, less conversion rate (16–18). A meta-analysis published by Wu et al. (19) and two prospective randomized studies of Badawy et al. (20) and of Singh et al. (21) show similar results for both techniques. In spite of longer operating time, retroperitoneal laparoscopy presents less ileus, limited spread of urine in case of leak, faster oral feeding, less need for post-operative analgesia and a shorter hospital stay. Recently, Kallas-Chemaly et al. (22) published the same benefits by retroperitoneal pyeloplasty for patients younger of 1 year of age. Finally, all authors agree that learning curve play an important role in the results and that the approach is dependent on surgeon's preference and experience.

If for older patients there is no doubt about the benefits and advantages of laparoscopy, for young children and infants, opinions are different. Tan (23) published in 1999 a series of 16 laparoscopic pyeloplasties from whom 2 of them, presented a recurrence. These 2 children were 3 month-old. His recommendation was not to use the laparoscopy for infants <6 months. Since then, several authors published their experience in contradiction with this opinion (2, 3, 24–28). Even Tan, few years later, changed his point of view and published with Cascio et al. (4) his new technique using “needlescopic” instruments introduced through the abdominal wall without a trocar and performing a “no touch” technique anastomosis without touching the ureteral or pelvic mucosa.

As mentioned previously the operating time in laparoscopic pyeloplasties decreased by time and is similar to open surgery in experimented hands. All our patients, in this age-group had been operated by consultants. However, minimal invasive approach can be taught to junior surgeons successfully, although it is a long learning process and remains a challenging task for a teaching center (15).

Robotic assisted surgery overcomes the difficulty of laparoscopy and retroperitoneoscopy facilitating suturing but there are two major limitations: the cost of consumables and the size of the ports for young children. In spite of these, Kutikov et al. (24) presented a successful series of robotic assisted laparoscopic pyeloplasties in 9 infants from 3 to 8 months of age with 100% success. Blanc et al. (29) support the advantages of robotic retroperitoneal pyeloplasties and good results with the last generation of Da Vinci Xi once the learning curve is finished. But their youngest patient was 2-year-old (12 kg). There is no doubt that robotic surgery makes pelvic-ureteric anastomosis easier for the surgeon.

The infants have a high sensitivity at CO_2_ effects, increased intra-abdominal pressure and hypothermia. A strict selection of patients associating other comorbidities is necessary in order to avoid any incident (30). There are several recent studies using near-infrared spectroscopy which showed a transient modification in cerebral oxygenation during the insufflation which increase with augmentation of the intra-abdominal pressure. Transient hypercarbia is ameliorated rapidly when CO_2_ insufflation is stopped (31, 32). Near-infrared spectroscopy also showed that in normal functioning kidney renal hypoxemia doesn't occur if age-appropriate insufflation pressure is respected and no problems of oliguria/anuria were noticed (33). Hypothermia and hypocarbia could be prevented by specific measures: low insufflation rate at about 6–8 mmHg (even stopped when necessary), shorter operating time, warming blanket, etc. Close collaboration between surgeon and anesthetist is necessary (31).

The antibiotic prophylaxis in antenatal hydronephrosis is a debatable subject. Several review and metanalyses tried to find an answer at ongoing discussion (34–37). They found out that the studies are difficult to be compared due to biases and lack of the same criteria of comparisons. Use of continuous antibiotic prophylaxis (CAP) in prenatal hydronephrosis has been based on low level of evidence. Even if CAP reduce the risk of UTIs in some children not all will benefit (32). Antibiotic prophylaxis during surgery was administered in all three departments. It was continued during stenting period only in one (where the percutaneous transanastomotic stent was inserted). In the other two departments (where double J was preferred) no prophylaxis was used.

Type of stenting is also a matter of debate. There are two principal options: percutanous transanastomotic drains which are kept in place for 12–14 days and are removed in outpatient clinic or double J stents which are withdrawn by cystoscopy 4–6 weeks after surgery.

Late complications rate (two patients −3.4%) are similar to the literature (2–4, 25, 27). Malrotated kidneys remain a challenge because of the unexpected position and technical difficulties, like in 1 of our cases. A caliceal-ureterostomy was not an option due to the thickness of the lower pole parenchyma nor did an anterior uretero-pelvic anastomosis because of kidney posterior rotation. In this case the follow-up from the second surgery is of only 1 year and the hypertension disappeared. More time is needed in order to be sure that the nephropexy is long term successful.

Our clinical series is, at our knowledge, the largest published in this age group with similar results and complications comparing to the ones already published (2–4, 25–28).

Generally, children who require surgery in the first months of life have severe UPJO obstruction, which is present since pregnancy and presenting mostly with decrease function of the affected kidney. The two patients drained during pregnancy, the two patients who required a nephrostomy before surgery and the one who had a spontaneous perforation demonstrate the potential seriousness of the disease. This could explain our observation that post-operatively there is a statistically significant reduction in the antero-posterior diameter of the pelvis and a slightly increase in renal function but not statistically significant. In grade IV hydronephrosis with parenchymal loss (38 of our patients, 63.4%), pyeloplasty is a salvage procedure. Our results show that minimally invasive surgery achieves this aim.

## Conclusions

Laparoscopic transperitoneal pyeloplasty is feasible and safe in children younger than 1 year of age. In recent years, laparoscopic approach has become progressively more standardized making this surgery easier. Nevertheless, it requires experience and good intra-abdominal suturing skills. Laparoscopy offers a very good view, shorter hospital stay, less post-operative pain and better cosmetic results. The advantages in comparison to the retroperitoneoscopy and robot-assisted laparoscopic pyeloplasty need to be determined by prospective randomized controlled trials. Open approach is no longer considered in the three centers included in this study.

In our society in constant evolution with increasing demands, requirements, and high expectations we need to adapt our techniques, preserving the old ones as basis of the new ones and for selected patients or rescue situations.

## Data Availability

The datasets for this manuscript are not publicly available because the patients' files are publicly available but information was collected for scientific purposes. Requests to access the datasets should be directed to Corina Zamfir, corina.zamfir@huderf.be.

## Author Contributions

CZ: data collection, wrote, and corrected the manuscript. ED and SV: data collection and manuscript preparation. ML, KK, SL, PP, and FV: performed surgery and revised the manuscript. HS: study idea, performed surgery, and revised the manuscript.

### Conflict of Interest Statement

The authors declare that the research was conducted in the absence of any commercial or financial relationships that could be construed as a potential conflict of interest.
